# Real-world evidence for coverage determination of treatments for rare diseases

**DOI:** 10.1186/s13023-024-03041-z

**Published:** 2024-02-07

**Authors:** Victoria W. Dayer, Michael F. Drummond, Omar Dabbous, Mondher Toumi, Peter Neumann, Sean Tunis, Nelson Teich, Shadi Saleh, Ulf Persson, Johann-Matthias Graf von der Schulenburg, Daniel C. Malone, Tay Salimullah, Sean D. Sullivan

**Affiliations:** 1 CHOICE Institute, School of Pharmacy, University of Washington, 1959 NE Pacific Street, Seattle, WA 98195 USA; 2 Centre for Health Economics, University of York, York, UK; 3 Novartis Gene Therapies, Inc., Bannockburn, IL USA; 4 Faculty of Medicine, Public Health Department, Aix-Marseille University, Marseille, France; 5 Tufts Medical Center, Boston, MA USA; 6 Rubix Health, Baltimore, MD USA; 7 Brazil Governance Network, Brasilia, Brazil; 8 American University of Beirut, Beirut, Lebanon; 9 The Swedish Institute for Health Economics, Lund, Sweden; 10 Institute for Risk and Insurance, Leibniz University Hannover, Hanover, Germany; 11 Department of Pharmacotherapy, University of Utah, Salt Lake City, UT USA

**Keywords:** Cell and gene therapy, Coverage determination, Coverage with evidence development, Health technology assessment, Rare disease, Real world evidence

## Abstract

Health technology assessment (HTA) decisions for pharmaceuticals are complex and evolving. New rare disease treatments are often approved more quickly through accelerated approval schemes, creating more uncertainties about clinical evidence and budget impact at the time of market entry. The use of real-world evidence (RWE), including early coverage with evidence development, has been suggested as a means to support HTA decisions for rare disease treatments. However, the collection and use of RWE poses substantial challenges. These challenges are compounded when considered in the context of treatments for rare diseases. In this paper, we describe the methodological challenges to developing and using prospective and retrospective RWE for HTA decisions, for rare diseases in particular. We focus attention on key elements of study design and analyses, including patient selection and recruitment, appropriate adjustment for confounding and other sources of bias, outcome selection, and data quality monitoring. We conclude by offering suggestions to help address some of the most vexing challenges. The role of RWE in coverage and pricing determination will grow. It is, therefore, necessary for researchers, manufacturers, HTA agencies, and payers to ensure that rigorous and appropriate scientific principles are followed when using RWE as part of decision-making.

## Introduction

In order for patients to gain access to new pharmaceuticals, manufacturers must successfully navigate two critical steps. First, they need to obtain marketing approval (i.e., approval to market) from a government regulatory body, such as the US Food and Drug Administration (FDA) or the European Medicines Agency (EMA). For this step, the regulatory agency requires proof of efficacy and safety, which are often obtained through the conduct of randomized, controlled trials. Many regulatory agency requirements and processes are similar across the globe. Some countries even allow the transfer of evidence packages from the EMA or the FDA to the approving country. The outcome of the regulatory review is a license to market the product under a specific set of conditions.

Regulatory approval is necessary, but not sufficient for patient access. Next, the manufacturer must obtain reimbursement or formulary listing from the relevant government or private sector payer(s). The evidence requirements and reimbursement processes for market access vary considerably by jurisdiction. Government-funded health technology assessment (HTA) bodies and private sector insurance companies control access. To gain coverage, these organizations use rigorous methods to assess the clinical and economic value of new treatments. In some cases, these organizations provide explicit methods and guidance for decision-making, including the use of real-world evidence (RWE) to supplement clinical evidence from the regulatory package. If, at the time of the initial coverage assessment, there are still uncertainties about the clinical evidence, the HTA body may grant conditional approval while additional evidence is gathered.

The generation and use of RWE has increased in recent times, partially in response to regulatory changes that encourage more rapid access to innovative treatments and HTA requirements for reimbursement. Regulatory bodies such as the FDA have recently issued guidance on the use of RWE to support labeled claims [1, 2], and the FDA notes in this guidance that the evidence packages submitted to support innovative treatments for rare diseases often lack comparative studies. The major HTA bodies have begun to revise their methods and guidance to encourage inclusion of RWE as part of the evidence submissions by industry, particularly when the regulatory evidence package is highly uncertain on relative effectiveness compared with therapeutic alternatives.

The overall purpose of this paper is to explore the myriad challenges associated with the generation and use of RWE for reimbursment determination of treatments for rare diseases, both during the initial coverage assessment and after HTA bodies have made conditional approval determinations.

## Background

Randomized, controlled trials (RCTs) are the gold standard for assessing comparative or relative effectiveness [3, 4]. RCTs are expensive, time-consuming, and often placebo-controlled rather than conducted with active comparators, making comparisons between therapeutic alternatives difficult. In addition, RCTs are often not reflective of actual clinical practice, in which conditions such as adherence, treatment duration, incentives, and outcome measures vary. In the case of rare disease treatments, RCTs may be difficult to conduct, either because of ethical considerations (in which the severity of disease may make the use of a placebo unethical), lack of sufficient funding, or the difficulties in recruiting sufficient patients. Therefore, the available clinical evidence on rare disease treatments will at best be based on small or short-term RCTs, or at worst based on single-arm observational studies. Though RCTs remain the primary standard for licensing approval, data from RWE studies are increasingly used to inform HTAs and pricing and reimbursement globally. There are still substantial barriers to widespread adoption of RWE studies by decision-makers as a primary source for evidence of clinical benefit [5].

RWE is evidence derived from real-world data (RWD) generated either prospectively or retrospectively from observations in clinical practice, using sources such as electronic health records (EHRs), insurance claims, mobile devices, or disease registries [6, 7]. Disease registries generally include patients diagnosed with a particular disease or patients utilizing a particular intervention. The registry can be populated from various sources such as case reports, EHR data, claims, or a combination thereof [8]. Registries are sometimes designed with a prospective study in mind, but other times exist for different purposes and can be repurposed for retrospective or prospective studies. RWE is especially important in the context of treatments for rare diseases, in which RCTs or other traditionally used interventional trial designs may be more challenging to conduct.

The landscape of decision-making by licensing agencies is continuously evolving, and as treatments become more specialized and personalized, the coverage and reimbursement decision criteria are also becoming further restricted to the population evaluated in the submitted evidence for the treatment being assessed. A strong view exists that RWE should and will play a more influential role in these decisions, and a number of HTA bodies have mentioned RWE within their general HTA guidelines [9–13]. To our knowledge, only three HTA bodies have published more detailed papers on RWE [12–14]. Recent guidance by the National Institute for Health and Care Excellence (NICE) represents one of the most detailed discussions of the issues in producing and using RWE [13], and the recent paper by Canada’s Drug and Health Technology Agency (CADTH) gives detailed guidance on the *reporting* of RWE studies [12]. However, RWE has not yet been featured as a topic in the deliverables in the European Network for Health Technology Assessment (EUNetHTA21) joint action (though one paper touches on it in the context of indirect treatment comparisons) [15, 16]. However, not only does the use of RWE for decision-making in the context of pricing, reimbursement, and coverage decisions vary based on the clinical situation being evaluated, but it also varies based on the context of the decision-maker.

RWE has primarily been used by licensing agencies for post-approval safety monitoring and to resolve uncertainties in the original data submitted. In reimbursement settings, RWE has been used in managed access/entry agreements (MAAs/MEAs) for estimating costs and benefits, and for monitoring compliance with prior authorization criteria or formulary status. Private payers in the United States often use RWE to inform formulary decisions and for assessing comparative effectiveness, particularly when head-to-head clinical trials are not available [17]. Though many guidelines recommend against replacing clinical trial evidence with RWE, and instead advise using RWE as a supplement to clinical trials, more recently, there is growing interest in the use of RWE for estimating relative clinical effectiveness in the initial approval, coverage, or reimbursement process, especially in rare diseases for which large, formal trials are not possible [1, 10, 12, 18, 19]. For instance, since 2020 in Germany, the Joint Federal Committee (Gemeinsamer Bundesausschuss, or G-BA) can require pharmaceutical companies to collect and evaluate data on medical products from clinical practice as part of the early benefit assessment. The prospective RWE study of onasemnogene abeparvovec in comparison with nusinersen, which started on February 1, 2022, is a recent example [20, 21].

Relative or comparative effectiveness assessment is defined as “comparing health-care interventions as used in practice to classify them according to their practical additional therapeutic value” [22]. The European Joint Scientific Consultation and Joint Clinical Assessment requirements are examples of recent initiatives in which RWE is envisioned as part of coordinated evidence dossiers for HTA decisions; however, the specific role of RWE for country-level decisions has not yet been made clear [13].

Given the growing use of RWE, it is important to consider the methodological and practical challenges involved, and to specify scientific best practice. This paper describes the challenges in generating and using RWE for reimbursement decisions for rare disease treatments and makes recommendations for how to respond to these challenges. We start with a detailed discussion of the considerations for and challenges of applying RWE approaches in rare medical diseases. We then put forward recommendations for researchers, analysts, and decision-makers who wish to apply and use RWE methods and data for rare diseases.

### Considerations for rare disease

For rare diseases in which an RCT may not be possible, RWE on the standard of care is sometimes used as a historical control [9, 23]. In situations in which waiting to capture true duration of effect would substantially delay patient access to treatment, RWE may be used to assess benefit after a conditional coverage or reimbursement decision is made, with plans to reassess the decision after a predetermined amount of time. This is known as coverage with evidence development, or CED [24]. In studies of rare disease treatments or precision oncology medicines, for example, trials for the initial regulatory approval decision may have reported a change in tumor response or a certain biomarker. Early patient access and reimbursement may be provided, but before making a final coverage determination, the payer may require a CED study to assess overall survival [18]. The initial RCTs in such cases may have demonstrated safety and initial response to support the preliminary coverage, but the CED studies conducted post-approval would serve to demonstrate longer term real-world effectiveness and value.

Alternatively, RWE may be requested by decision-makers to compare the treatment of interest with the current standard of care or another comparator that may not have been included in the RCT for regulatory or initial coverage purposes, such as the prospective RWE study requested by the G-BA mentioned above. Jurisdictions using RWE to inform decisions must weigh the implications of delaying care versus the benefit of granting coverage with evidence development, knowing that there is the possibility that the decision may need to be reversed based on the longer-term results, a process which may be difficult [24, 25].

In studies without randomization, there is substantial potential for bias, and study design (cohort, case-control, cross-sectional, etc.) may prohibit causal conclusions [6, 22]. Retrospective studies using databases, EHRs, or registries may be limited by incomplete data and reporting bias; prospective studies may allow for greater control over data collection but may be restricted by feasibility and funding, which is often further limited in rare diseases [1, 10].

RWE is likely to become more relevant and common as more cell, gene, and other rare disease therapies that are more challenging to evaluate by traditional means are developed and as decision-makers become more receptive to RWE [26]. Thus, RWE can allow for earlier access to treatments, provide insight into practicality or clinical gaps that randomized trials may overlook, and identify specific patient populations for which a treatment is most beneficial. However, there are substantial barriers and limitations surrounding study design, role of the decision-maker/regulator, reporting requirements, funding arrangements, and evaluation that are amplified in the context of rare diseases.

### Challenges in generating comparative RWE

#### Selection bias, confounding, and use of multiple clinical sites

Historically, the most common and challenging methodological issues when generating RWE are selection bias and confounding [6, 22]. This is compounded when the disease of interest is rare, because of the small and heterogeneous nature of the patient population. One example of selection bias is when patients who seek out and enroll in a study or registry are systematically different (either sicker or healthier, more likely to be adherent, etc.) from those who do not enroll, thus limiting the application and generalizability of the study results.

In comparative RWE studies, a lack of randomization increases the likelihood of confounding because of differences between the groups being compared, and there may be unavoidable differences in treatment patterns (including frequency and characteristics of follow-up visits), discontinuation, and misclassification [22]. In rare diseases, implementing extensive inclusion criteria may severely limit the sample size and is thus not feasible, particularly since the patients included may be more heterogeneous. Further, generalizability and ensuring that the decision is relevant to the patient population with the disease for which coverage is desired may be difficult, and it may be impossible to define patient subgroups for reimbursement purposes.

If a comparative study (whether RWE or an RCT) is being designed for a hypothetical rare disease, the enrolled patients may be from a registry, a single clinic, or even from just one clinical specialist. Recruiting and enrolling patients from these sources increases the risk of excluding those who remain undiagnosed, those who may not have access to care logistically or financially, those whose disease was so severe that they are no longer living, those whose treatment was conducted at a site or system where a differing treatment practice may affect outcomes, and those who have given up trying to treat their condition. These patients who were not included in the study must be acknowledged and accounted for, because outcomes in the broader covered population will likely differ from the patients enrolled in the study in various, likely unknown, ways.

Bias and confounding can be addressed in both the study design and in the analytic/statistical methods. The ISPOR Good Research Practice Task Force on Prospective Observational Studies’ report on assessing comparative effectiveness recommends first that studies are designed with key policy questions in mind, with purpose and hypotheses clearly stated, and that strategies (e.g., inception cohorts, new user designs, multiple comparator groups, matching designs, and assessment of outcomes thought not to be impacted by the therapies of interest) be considered for identifying and adjusting for unmeasured confounding [22]. However, feasibility constraints are substantial for rare diseases, so these strategies are often difficult in such contexts. In the statistical model used for analyses, confounders should be carefully identified and controlled for, if possible, and sensitivity analyses should be conducted to strengthen and demonstrate the robustness of results. Additional analytic strategies proposed by the ISPOR Task Force on Good Research Practice include stratification, multivariable regression, matching methods, propensity scoring, instrumental variable approaches, and other structural modeling techniques [22]. However, use of these analytic approaches is often limited for rare diseases because of small sample sizes.

In many RWE studies, it is necessary to utilize multiple clinical sites to implement a study protocol, and this is especially true for rare diseases with few patients in any given geography. Large RCTs and observational studies may also use multiple clinical sites but will generally have several patients at each site to help identify between-site differences. In RWE studies of rare diseases, the number of patients at each site may be very few. In addition to the potential administrative burden related to greater degrees of communication and coordination required, or data quality issues related to differences in documentation methods, individual clinic-related factors and population differences related to geography, urbanicity, provider preferences, and study recruitment practices may result in confounding that is difficult to identify and statistically control.

#### Use of historical/external controls

In rare and severe diseases, a historical control or external control from a previous trial, modeled natural history of the disease, observational studies, or real-world retrospective or concurrent data may be used as the control arm, because of ethical concerns surrounding the assignment of patients experiencing a debilitating disease to a placebo arm [8, 27]. Regulatory agencies generally discourage the use of historical/external controls, but exceptions are increasingly observed in regulatory approvals. The FDA has indicated that exceptions are acceptable when the disease is rare and serious with a substantial unmet medical need, the disease course is measurable and well documented, and the study population and historical control are comparable [28]. It must be noted that diagnostic procedures and standards of care vary and change over time, so the selection of the historical control should account for these elements.

In the United States, some therapies for rare diseases have been approved based on a single-arm trial compared with a historical control, whereas others are granted accelerated approval based on a single-arm study with a historical control and a requirement to conduct a randomized trial. Cerliponase alfa, for example, was granted full approval for a specific form of Batten disease in children based on a single-arm trial compared with a historical control from a natural history registry in 2017 [8, 29, 30]. Blinatumomab, however, was given accelerated approval status in 2014 for the treatment of Philadelphia chromosome–negative relapsed or refractory B-cell precursor acute lymphoblastic leukemia based on a single-arm trial compared with historical data, but a randomized trial was required. Full approval was granted in 2017 based on the results of the phase 3 TOWER trial [31, 32].

Several pivotal trials using a historical control in their studies have been conducted for advanced (cell and gene) therapies (e.g., tisagenlecleucel, axicabtagene ciloleucel, autologous CD34 + cells transduced to express ADA [Strimvelis], onasemnogene abeparvovec, elivaldogene autotemcel, and idecabtagene vicleucel), and these were the basis for regulatory decisions in multiple countries [9]. However, these approvals have often required the manufacturer to conduct prospective observational studies after approval for long-term safety and efficacy, if not also an RCT. Globally, many regulators are still hesitant to accept single-arm trials in general, which may delay access to some rare disease therapies.

Use of historical/external controls becomes more problematic when addressing the question of comparative/relative effectiveness in a reimbursement setting. Onasemnogene abeparvovec and nusinersen, for example, are two treatments for spinal muscular atrophy (SMA). Pivotal trials for nusinersen were two randomized, double-blind, sham-procedure controlled studies (one for infantile SMA and one for late-onset SMA) and one open-label uncontrolled trial for presymptomatic SMA [33]. Approval for onasemnogene abeparvovec, however, was based on an open-label, single-arm trial of 21 patients compared with a historical control [34, 35]. The systematic differences between the clinical assessments and the populations in the clinical trials for the two products makes comparison very challenging, but indirect treatment comparisons have been published [36, 37]. The health technology appraisal institute IQWiG in Germany has determined that, despite differences in regulatory-approved target populations, separate benefit assessments, and published indirect treatment comparisons for onasemnogene abeparvovec and nusinersen, an RWE study is required to assess their comparative effectiveness using a global patient registry. Development of this RWE study requires substantially more investment and capability development than first anticipated by the manufacturers. In addition, the study must be designed to detect very large treatment effects to demonstrate comparative effectiveness because of all the potential sources of bias associated with the use of observational data [9, 20].

#### Outcome selection & surrogate or intermediate endpoints

As in RCTs, a real-world comparative study must also have clear definitions of the outcomes that are measured, but certain outcomes pose difficulties in the context of RWE because of feasibility or measurement constraints; for example, clinic visits may be less frequent than in an RCT, and outcome measurements need to be such that routine and accurate collection in a standardized manner in medical practice is possible. For rare diseases, there may be further constraints on this depending on the outcome instrument, because the choices of data sources may be further limited, and there may not be widely accepted standards or clear guidelines for outcome measurements in the disease of interest. For example, an ISPOR Task Force on outcome measurement in clinical studies of rare disease treatments argues that there are a number of additional challenges because of the small patient population and heterogeneity of the condition or study sample. Also, few disease-specific patient reported outcome measurement instruments for rare diseases exist [38].

Endpoints such as pain are subjective and should be selected with caution. Subjective endpoints can vary substantially in how they are measured and assessed, and thus are subject to considerable uncertainty, whereas objective endpoints, such as death or hospitalization, can be more easily captured [1]. Digital health tools, which can assist in collecting both subjective and objective information, are increasingly being used to monitor the outcomes of studies and can be an option for individual health outcome measurements in prospective studies [39].

Surrogate or intermediate endpoints are outcomes used in place of directly measuring clinical benefits and may be useful in situations in which the clinical benefit may take a long time to observe or is very difficult to measure [40]. Surrogate or intermediate endpoints are common in rare and chronic diseases to allow for shorter follow-up and smaller sample sizes, both in RCTs and in RWE studies [9]. For example, voretigene neparvovec-rzyl is a gene therapy for retinal dystrophy, and the manufacturers created a novel endpoint called the “multi-luminance mobility test (MLMT) course” for their phase 3 trial to test patients’ vision [9, 41].

Surrogate or intermediate endpoints must be validated to confirm that they are an appropriate proxy for clinical benefit, which is difficult in rare diseases where sample sizes are small and validation guidelines may not exist. If studies have already been conducted in the disease of interest, however, there may be a precedent for acceptable surrogate endpoints [40]. In comparative studies, the surrogate endpoint must be equally relevant to the effects of both interventions. Surrogate or intermediate endpoints in RWE also need to be an outcome that is measured in the course of routine practice, as a manufacturer-created test such as the MLMT would not be something that health care providers are regularly monitoring in their patients.

When selecting outcomes of interest, statistical significance is of concern, but consideration should also be given to clinical importance and the research question of interest. The minimal clinically important difference (MCID) can be difficult to determine, particularly in rare diseases in which effect sizes may not be precise because of small samples and population heterogeneity, and values in the literature may be sparse [22]. Regulatory bodies may also differ in their standards for MCID selection. Thus, justification for choice of outcome and explanations surrounding clinical importance should be clearly defined, starting with the research question that the study is seeking to answer.

#### Length of study

RWE is often considered as a means of filling evidence gaps, particularly in the context of reimbursement decisions, perhaps because of a need for long-term evidence or for evidence in patient populations that was not captured in the primary regulatory studies. When designing studies, however, the length of the study has considerable implications for feasibility. Longer studies have increased drop-out rates and loss-to-follow-up over time but are often necessary in cases in which evidence of long-term effectiveness, durability of treatment effect, and safety is needed. This is particularly true for RWE that may be based on more passive data collection or less frequent follow-up than a formal trial. Rare diseases are often chronic and/or have slow development and require longer follow-up, but the drop-out rates over the duration of the studies threaten the results because of the already low sample sizes of rare disease RWE studies. One approach to shortening the duration of a study for the purpose of a licensing decision is to use surrogate endpoints. However, because of the need to make subsequent reimbursement decisions, careful consideration must be given to the selection of such endpoints, as discussed above.

#### Data quality

Registry, health insurance claims, EHR, and RWD in general all have the potential to be lacking in data quality because of missing or incomplete information that exposes the data to information bias [10, 13, 42]. Selection of an appropriate data source for the outcome(s) of interest is an obvious consideration, as claims data include pharmacy fills and encounters with providers but do not include laboratory results, whereas EHR chart reviews may be more complete but vary in terms of quality and standardization and do not necessarily track patients across providers [10, 43]. Registry data should be up-to-date and an appropriate reflection of current practice, and if the registry was initially intended for a particular purpose and is being repurposed for a particular study, data analysts should ensure that this repurposing is possible and appropriate. Incompleteness of data may also necessitate linking between sources, if multiple sources are required for the study of interest. Data quality monitoring in RCTs is routine and expected; however, this is not the norm for RWE. Study sponsors often direct resources toward recruitment and follow-up and neglect source data verification, which can result in undetected problems with the data used [22]. Data quality has become a high priority for all decision-makers, indicating that reliability of registry data needs to be ensured and documented for transparency purposes [1, 5, 44]. Various groups have provided guidance for evaluating data quality in RWE (examples include EUnetHTA’s REQueEST tool for registries, IQWiG’s guidance on analysis of routine practice data, and ISPOR’s Task Force on Retrospective Databases) [5, 7, 42, 45]. Emerging methods using machine learning and/or artificial intelligence may be considered to monitor the quality of data collected.

#### Practical issues with evidence generation requirements (such as CED and MEA schemes)

In the United States, the Centers for Medicare & Medicaid Services (CMS) has a designation of CED, in which CMS may determine that coverage for a drug or device is limited to the context of a clinical study, with the intent to revise the coverage decision based on the results of the studies conducted [46–52]. The requirement for data collection is often centered around medium- or long-term evidence compared with the premarket trials or comparative effectiveness in the Medicare population [49]. A study examining 26 CED programs between 2005 and 2021 found that program duration varied substantially (ranging from 1 to 16 years), and many failed to provide evidence that was useful to modify the initial coverage decision, with only three retiring the requirement and two revoking coverage. In addition, this designation has rarely been used for pharmaceuticals in the United States, although this may change with the advent of gene therapies and other medicines with potential long-term benefits. In 2022, aducanumab, indicated for the treatment of Alzheimer’s disease, was granted CED designation, which limited Medicare coverage of the drug to individuals who were participating in CMS-approved studies. The use of RWE in this context is that “registry data may be used to assess whether outcomes seen in carefully controlled clinical trials (e.g., FDA trials) are reproduced in the real-world and in a broader range of patients” [50]. Thus, RWE in this case is being used to support data from previously conducted controlled clinical trials rather than to inform an initial decision.

In Europe, RWE forms a key part of MEAs that are agreed upon between payers or pricing and reimbursement agencies and manufacturers to enable the entry of new drugs and other health technologies into the health care system. CED and MEAs are similar, in that a technology is given a conditional reimbursement decision based on a commitment to collect more data, to mitigate either financial or clinical uncertainty [53, 54]. Kang and Cairns discuss the process for cancer drugs in England, in which, after an initial appraisal decision not to recommend routine commissioning, treatments are made available through the Cancer Drugs Fund (CDF) and are re-appraised using additional clinical data (including RWD) to address uncertainty present in the initial appraisal [53]. However, their study found that RWD played a limited role in addressing uncertainty in reassessments to date, indicating the opportunity for improvements in data collection (longer follow-up in particular) for RWD to be more informative in this process [53]. Facey et al., report on performance (outcomes)-based MEAs for two rare disease treatments (nusinersen and tisagenlecleucel) in Australia, Canada, and countries in the European Union [54]. Depending on the treatment and the country, data were either collected from existing registries, by establishing new registries, or through other forms of prospective studies.

Because conditional reimbursement implies that all eligible patients should have access to therapy, it may be necessary to set priorities for patient selection for inclusion in the study. However, Facey et al., point out that for rare diseases it may be valuable and feasible to study all patients [54]. Given the heterogeneity in the patient population, both patient-level and patient-reported data are important, and several of the MEAs studied planned to collect patient-reported outcomes, which are particularly important in rare diseases.

Another concern with an evidence generation requirement is the role played by each stakeholder. If a payer requires a study to be conducted for reimbursement purposes, the roles of the payer, manufacturer, health care providers, and patients in the study design, data collection, and funding should be clearly defined [24]. Facey et al., argue that “the jury is still out” as to whether MEAs can be successful for rare disease treatments, but “perhaps through greater collaboration among HTA/payers and with stakeholders, there can be more transparency about uncertainties that exist, constructs for data collection, and sharing of results that will optimize treatment and improve health service efficiency” [54]. They point out that if MEAs are to be a credible reimbursement route for rare disease treatments, the costs and feasibility of collecting sufficient data to inform decisions must be scrutinized from the outset, and steps must be taken to ensure data quality and completeness. As part of an EU-funded research project, Facey et al., have developed guidance on the use and implementation of outcomes-based managed entry agreements for rare disease treatments [55].

#### Generalizability and reproducibility

The practical issues within a jurisdiction are compounded when considering applications *between* jurisdictions. Different payers and HTA bodies have different approval requirements, timelines, and levels of involvement, and their reimbursement schemes and populations may also differ considerably. Some jurisdictions have published RWE frameworks and guiding principles that indicate slightly different uses and applications of RWE in decision-making between jurisdictions, meaning that a study designed to support a decision in one country may not necessarily be used to support that decision in a different country [1, 13, 19, 31]. If an RWE study is designed in collaboration with a given reimbursement body or payer for approval purposes, how can the results of that study then be applied to another jurisdiction?

Tisagenlecleucel and axicabtagene ciloleucel, both chimeric antigen receptor T-cell (CAR-T) therapies, are undergoing RWD collection in various countries for the purpose of eventual reassessment of reimbursement and coverage decisions. In France, both therapies have been approved, conditional upon the establishment of a CAR-T therapy registry for RWD collection to allow for annual evaluation and eventual reassessment of clinical benefit [56, 57]. Similarly, in England, NICE approved tisagenlecleucel and axicabtagene ciloleucel for patient access through the CDF, conditional on gathering additional RWE data (primarily follow-up data from the clinical trials but supported by RWE from various datasets) for future price reassessment after 5 years. In Scotland, both were approved under the ultra-orphan pathway with an end-of-life modifier, and with the requirement that further clinical effectiveness data be submitted for reassessment after 3 years [56, 57]. However, lack of standardization of data collection across countries leads to skepticism between decision-makers about the acceptability of results [4]. Given this concern and the differing requirements between countries, once data are collected (including via registries), it is difficult to know whether results will be acceptable in other jurisdictions. This also may cause issues with data privacy, as some jurisdictions require access to source data. Data ownership and privacy laws would need to be considered [19].

### Practical suggestions for real-world study designs for reimbursement decision-making

There are several useful and pragmatic approaches to designing and conducting RWE studies. Equally, there are many contexts in which RWE can be used and various questions these data can be used to answer. Thus, guidance for RWE should not be overly prescriptive [43]. Here, we present a list of suggestions for consideration when developing RWE studies for assessing comparative effectiveness in the context of a rare disease for the purpose of coverage or pricing determinations. Although not all suggestions can be followed in every case, those designing and conducting RWE studies should seek to follow them, as they will help to reduce the concerns of payers and HTA bodies. The suggestions and the challenges each suggestion helps address are summarized in Table 1.

**Table 1:** Challenges & practical suggestions

Challenge	Details	Suggestions
Selection bias, confounding, and use of multiple clinical sites	• These challenges lead to limited generalizability for informing decisions • Rare diseases often have small, heterogeneous, and limited patient populations • Typical bias mitigation strategies are often limited in rare diseases because of feasibility constraints	• Consider how patients will be recruited into the study • Acknowledge and account for the main source of bias • Determine the statistical methods for adjusting for bias
Historical/ external controls	• Historical cohorts are often used as the control arm in rare disease trials • Many entities grant approval or coverage based on these comparisons • These are problematic for addressing questions of comparative/relative effectiveness	• Be clear on the intended use of the RWE • Decide on study design considerations • Consider how patients will be recruited into the study
Outcome selection and surrogate endpoints	• Rare diseases may lack standards for outcome measurements • MCID can be difficult to determine in rare diseases because effect sizes may not be precise and values in literature may be sparse • Surrogate endpoints can be useful but can be difficult to validate in rare diseases	• Identify appropriate outcomes and consider measurement practicality • Assess the overall feasibility of the study • Start with the research question of interest when selecting outcomes
Length of study	• RWE is often considered as a means of filling evidence gaps, perhaps due to a need for long-term evidence • Length of study for rare diseases that are often chronic and/or have slow symptom onset may be limited by feasibility, cost, and risk of withdrawal	• Consider the overall length of the RWE study • Identify appropriate outcomes and consider measurement practicality
Data quality	• RWE data are collected from registries, health insurance claims, EHRs, and other forms of data, all of which have potential for missing or incomplete information • RWE data is often collected for one reason and re-purposed for another • Incompleteness of data may necessitate linking between sources • Data quality issues can result in information bias	• Determine how data quality will be monitored
Practical issues	• There are currently no global standards for use of RWE by payers • Roles and incentives of various stakeholders can be difficult to align • MEA and CED schemes may be difficult to implement for rare diseases due to significant methodological and implementation challenges	• Assess the overall feasibility of the study • Be clear on the intended use of the RWE • Clearly define stakeholder roles, investments, and involvement
Generalizability & reproducibility	• Practical issues within jurisdictions are compounded when considered between jurisdictions • Differing global requirements may prohibit or limit use from country to country, which can result in additional required studies and further costs if studies are not generalizable	• Consider the points listed above in the context of each country of interest

*CED* coverage with evidence development, *EHR* electronic health record, *MEA* managed entry agreement, *MCID* minimal clinically important difference, *RWE* real-world evidence

#### Be clear on the intended use of the evidence

There are many potential uses of RWE, including the assessment of long-term benefits and risks and the performance of therapies outside of demonstrating efficacy. The assessment of relative clinical effectiveness is the most challenging use of RWE and the most subject to potential bias. Prespecified clarity around the specific purpose and intended use of the RWE study will help guide research questions, study objectives, study design and statistical evaluation, and interpretation of results.

#### Decide on study design considerations in light of the study purpose

Decide on the question to be addressed based on the intended use of the RWE and whether it will impact the reimbursement decision. For example, if alternative treatments are being compared, decisions will be required on appropriate comparators, the MCID being sought, and the degree of uncertainty considered acceptable. These decisions will influence sample size, comparator selection (such as a historical/external control), and possibly the length of the study.

#### Consider the overall length of the RWE study

The length of the study should be determined by the hypothesis being tested. However, the longer the study continues, the harder it will be to maintain the interest and commitment of study participants and those who make decisions based on the study results. The overall length of the study should be considered in this context.

#### Identify appropriate outcomes and consider measurement practicality

What outcomes will be collected in this study? Are these outcomes regularly collected in practice? Are there substantial barriers for the participating clinics/hospitals in the course of data collection, such as high costs associated with integration into a registry? Are the selected outcomes clinically important and do they have a prespecified MCID? Are the selected outcomes acknowledged and accepted by the decision-maker concerned? Are subjective endpoints reliable and informative? Are any surrogate or intermediate endpoints being used, and if so, have they been validated? Accepted and validated outcomes instruments should be used when possible, and justification for the choice of outcomes and explanations surrounding clinical importance should be clearly defined. Ensure that in a comparative study, the type of benefits assessed are the same between the two treatments, while considering that different treatment options for the same disease may have different treatment targets.

#### Consider how patients will be recruited into the study

Bias can be introduced if the patients recruited to the study are not representative of the patient population. Will it be mandated that all patients eligible for treatment be enrolled in the study? If not, will there be clear inclusion and exclusion criteria? Is there a good understanding of how included and excluded patients differ? How much discretion will be given to clinical centers participating in the study? Should each site have a minimum number of patients to recruit? If a historical or external control is being used, are patient features and standards of care similar to those in the study population?

#### Acknowledge and account for the main sources of bias

Is there potential selection, measurement, maturation, instrumentation, or regression to the mean bias? How will patients be allocated to the treatments being examined in the study? If the allocation is not random, what criteria will be applied? Will some clinical centers only treat patients with one treatment? Will measurements of outcomes be standardized across centers? How will normal growth/improvement be separated from treatment effects? Were patients in one group selected based on having extreme values on an outcome of interest as compared with patients in other groups? Have other sources of bias been identified and accounted for? Will accounting for such biases affect the study objective? How might results and conclusions be affected?

#### Determine the statistical methods for adjusting for bias

In a non-randomized study, it is likely that the patients receiving alternative therapies will be different in terms of their clinical characteristics. How will selection bias be addressed in the design and/or analysis? The options include matching approaches, such as propensity scoring, or multivariate regression, with or without the use of instrumental variables. Use of instrumental variables is encouraged, when possible, because this method can adjust for unobserved biases in many instances.

#### Determine how data quality will be monitored

Due to their nature, RWE studies are more difficult to monitor. Will the data required be easy to collect in a real-world setting, and what efforts can be made to minimize withdrawal from the study and to follow patients who do withdraw? Are the responsibilities for data collection clear, and is there adequate funding? Source data verification should be performed, and data source reliability should be established and documented for transparency.

#### Assess the overall feasibility of the study

RWE studies require considerable effort to conduct. Make an overall assessment of the feasibility of the study based on these factors: (1) the difficulty of answering the question that is being posed, (2) the likelihood of stakeholder support, and (3) the time and resources required.

#### Ensure alignment of stakeholder roles and incentives

Due to the many stakeholders involved in RWE generation and use in decision-making, stakeholder involvement and incentives should be clearly defined early in the study design process. Who is funding the study? How much time are stakeholders putting into the design and review process? What is each stakeholder’s role in study design, monitoring/maintaining data quality, and analysis? Will any stakeholders have access to the data and the ability to use it in the future?

## Conclusions

The role of RWE in regulatory processes, label considerations, and coverage and pricing determinations will grow. Manufacturers should be encouraged to pursue a rigorous RWE strategy at the time of investing in their pivotal programs. Careful attention should be paid to the myriad methodological challenges faced when designing, conducting, and evaluating RWE studies, especially in rare diseases. It is, therefore, incumbent upon manufacturers, researchers, and HTA agencies to assure that rigorous and appropriate scientific principles are followed when applying RWE as part of coverage and pricing determinations.

## Data Availability

All data generated or analyzed during this study are not hosted online, but are included in this publication and available from the authors under reasonable request.

## Abbreviations

CADTH: Canada’s Drug and Health Technology Agency
CAR-T: chimeric antigen receptor T-cell
CDF: Cancer Drugs Fund
CED: coverage with evidence development
CMS: Centers for Medicare & Medicaid Services
FDA: US Food and Drug Administration
EHR: electronic health record
EMA: European Medicines Agency
EU: European Union
EUnetHTA: European Network for Health Technology Assessment
G-BA: Gemeinsamer Bundesausschuss
HTA: health technology assessment
MAA: managed access agreement
MEA: managed entry agreement
MCID: minimal clinically important difference
MLMT: multi-luminance mobility test
NICE: National Institute for Health and Care Excellence
RCT: randomized controlled trial
RWD: real-world data
RWE: real-world evidence
SMA: spinal muscular atrophy
